# Autoantigen microarrays reveal myelin basic protein autoantibodies in morphea

**DOI:** 10.1186/s12967-022-03246-5

**Published:** 2022-01-24

**Authors:** Jane L. Zhu, Ricardo T. Paniagua, Henry W. Chen, Stephanie Florez-Pollack, Elaine Kunzler, Noelle Teske, Yevgeniya Byekova Rainwater, Quan-Zhen Li, Gregory A. Hosler, Wenhao Li, Denise M. O. Ramirez, Nancy L. Monson, Heidi T. Jacobe

**Affiliations:** 1 Department of Dermatology, University of Texas Southwestern Medical Center, 5323 Harry Hines Blvd., Dallas, TX 75390-9069 USA; 2 Department of Immunology and Microarray Core Facility, University of Texas Southwestern Medical Center, Dallas, TX USA; 3 ProPath, Dallas, TX USA; 4 Department of Neurology and Neurotherapeutics, UT Southwestern Medical Center, Dallas, TX USA

**Keywords:** Myelin basic protein, Morphea, Antibody

## Abstract

**Background:**

Morphea is an autoimmune, sclerosing skin disorder. Despite the recent emphasis on immune dysregulation in morphea, the role of autoantibodies in morphea pathogenesis or utility as biomarkers are poorly defined.

**Methods:**

Autoantigen microarray was used to profile autoantibodies from the serum of participants from the Morphea in Adults and Children (MAC) cohort. Clinical and demographic features of morphea patients with myelin basic protein (MBP) autoantibodies were compared to those without. MBP immunohistochemistry staining was subsequently performed in morphea skin to assess for perineural inflammation in areas of staining. Immunofluorescence staining on mouse brain tissue was also performed using patient sera and mouse anti-myelin basic protein antibody to confirm the presence of MBP antibodies in patient sera.

**Results:**

Myelin basic protein autoantibodies were found in greater frequency in morphea (n = 50, 71.4%) compared to systemic sclerosis (n = 2, 6.7%) and healthy controls (n = 7, 20%). Patients with MBP antibodies reported pain at higher frequencies. Morphea skin biopsies, highlighted by immunohistochemistry, demonstrated increased perineural inflammation in areas of MBP expression. Immunofluorescence staining revealed an increased fluorescence signal in myelinated areas of mouse brain tissue (i.e. axons) when incubated with sera from MBP antibody-positive morphea patients compared to sera from MBP antibody-negative morphea patients. Epitope mapping revealed target epitopes for MBP autoantibodies in morphea are distinct from those reported in MS, and included fragments 11–30, 41–60, 51–70, and 91–110.

**Conclusions:**

A molecular classification of morphea based on distinct autoantibody biosignatures may be used to differentially classify morphea. We have identified anti-MBP as a potential antibody associated with morphea due to its increased expression in morphea compared to healthy controls and systemic sclerosis patients.

**Supplementary Information:**

The online version contains supplementary material available at 10.1186/s12967-022-03246-5.

## Background

Morphea, also known as localized scleroderma, is an autoimmune disorder in which inflammation gives way to excessive collagen deposition leading to dermal and/or subcutaneous sclerosis. Morphea initially appears as active, inflammatory skin lesions characterized by a dense dermal and subcutaneous lymphocytic infiltrate, manifesting clinically as erythema and edema [1]. A fibrotic damage phase follows, characterized by closely packed homogeneous dense collagen deposition manifesting as fibrotic patches or linear bands of skin that are thick, hard, and discolored [2]. Fibrosis and resultant atrophy of the skin, underlying connective tissue, and bone cause deformity and severe functional impairment [1–4]. Despite the recent emphasis on immune dysregulation in morphea, the pathogenesis of morphea remains poorly understood and little is known about autoantibodies associated with morphea..

Studies to date imply that B cells and autoantibodies may play a role in morphea pathogenesis. For example, plasma cells are present in morphea lesions, composing the second most common cell type after lymphocytes [5]. Several potential autoantibody associations have been described in subsets of morphea patients including anti-histone, anti-topoisomerase IIα, anti-U3-small-nuclear-ribonucleoprotein antibody (U3-snRNP), anti-endothelial cell, anti-matrix metalloproteinase 1, and anti-Th/To ribonucleoprotein, among others [4, 6–13]. Although these studies suggest the possibility that these autoantibodies may be important for pathogenesis or as biomarkers, their role is poorly understood.

We undertook this study to identify autoantibodies associated with morphea and determine the association of these antibodies with specific clinical and demographic features of the disease. We used the resources of the Morphea in Adults and Children (MAC) cohort, which allowed us to determine autoantibody profiles in a cohort of patients with corresponding, well-annotated demographic and clinical features in comparison to matched healthy controls.

## Materials and methods

### Study participants

Morphea cases were obtained from the MAC cohort, which contains 346 adults (age ≥ 18 at enrollment) and 107 children (age ≤ 17 at enrollment) with morphea at the time of this study. All patients or guardians provided written consent for study inclusion, which was approved by the University of Texas (UT) Southwestern Medical Center Institutional Review Board (IRB). The MAC cohort is a prospective registry designed to better understand the demographic, clinical, and autoimmune features of morphea. Criteria for enrollment and source of patients in the MAC cohort have been previously published [1]. Inclusion criteria for the present study included: enrollment in the MAC cohort; sufficient sera for analysis; availability of demographic and clinical outcomes; and no exposure to systemic immunosuppressives for 3 months preceding enrollment. A total of 70 morphea patients met inclusion criteria.

Non-morphea study participants were obtained from three disease registries: (1) Systemic sclerosis cases were obtained from the Genetics versus Environment in Scleroderma Outcomes Study (GENISOS) conducted at the University of Texas Medical Branch at Galveston (UTMB), the University of Texas Health Science Center at Houston (UTHSC-H), and the University of Texas Health Science Center at San Antonio (UTHSC-SA) (n = 30) [14]. (2) Multiple sclerosis cases were obtained from the UT Southwestern Clinical Center for Multiple Sclerosis (n = 6). (3) Control cases were obtained from the Division of Rheumatology at the UTHSC-H (n = 35) [15]. All healthy control cases were screened for a personal or family history of any autoimmune diseases and were excluded if they reported having a relevant history. All controls were age-, gender-, and ethnicity-matched to morphea cases, and selection criteria were similar to those previously selected in a study by Arnett et al. [15]. All registries received IRB approval at their participating institutions. Serum samples obtained from collaborating registries were de-identified. All samples were processed using the same protocols.

### Assessment of disease severity, activity and clinical parameters

All morphea participants were evaluated and assigned a clinical severity score and assessed for the presence of functional impairment, which was defined as the presence of limited range of motion, contractures, and/or limb length discrepancy due to morphea involvement. Patients in the MAC cohort were evaluated and scored by a single investigator (H.J.) using the Localized Scleroderma Cutaneous Assessment Tool (LoSCAT) [16]. The LoSCAT is divided into the modified Localized Scleroderma Skin Severity Index (mLoSSI) and the Localized Scleroderma Skin Damage Index (LoSDI). The Physician Global Assessment of Activity (PGA-A) and Damage (PGA-D) were scored as part of the LoSSI and LoSDI, respectively. The validation of these clinical measures has been previously reported [1]. At the time of the visit, each patient was asked to score the severity of pain and itch in their lesions on a scale of 1–10. Pain and/or itch was considered present if the score was greater than 1.

### Autoantigen arrays

Candidate autoantigens (including MBP) presented in the arrays were associated with either autoimmune cutaneous diseases or systemic diseases based on previously published studies [1, 17, 18]. One hundred and twenty peptides and proteins representing putative cutaneous autoantigen epitopes were printed in duplicates onto Nitrocellulose film slides (Grace Bio-Labs). Patient serum samples (2 uL) were pretreated with DNAse-I and then diluted 1:50 in PBST buffer for autoantibody profiling. The diluted serum samples were incubated with the autoantigen arrays, and autoantibodies binding with arrayed proteins were measured with cy3-conjugated anti-human IgG (1:1000, Jackson ImmunoResearch Laboratories) and cy5-conjugated anti-human IgM (1:2000, Jackson ImmunoResearch Laboratories), using a Genepix 4200A scanner (Molecular Device) with laser wavelength of 532 nm and 635 nm. The resulting images were analyzed using Genepix Pro 7.0 software (Molecular Devices). The median of the signal intensity for each spot was calculated and subtracted the local background around the spot, and data obtained from duplicate spots were averaged. The background subtracted signal intensity of each antigen was normalized to the average intensity of the human IgG or IgM, which were spotted on the array as internal controls. Finally, the normalized fluorescence intensity (NFI) was generated as a quantitative measurement of the binding capacity of each antibody with the corresponding autoantigen. Signal-to-noise ratio (SNR) was another quantitative measurement of the true signal above background noise. SNR values equal to or greater than 3 were considered significantly higher than background, and therefore true signals. The NFI of each autoantibody was used to generate heatmaps using Cluster and Treeview software (http://bonsai.hgc.jp/~mdehoon/software/cluster/software.htm). Statistical analysis was performed using R to identify differential expressed antibodies between different study group.

### Immunoassays

Indirect ELISAs were performed to measure MBP peptide autoantibody reactivity. MBP peptides were custom-synthesized using 20-amino acid overlapping sequences as previously described (Van Haren et al., 2013). Nunc-Immuno Maxisorp 96-well plates (Sigma-Aldrich) were then incubated overnight at 4 °C with MBP peptides (Sigma-Aldrich PEPscreen) at 5 µg/ml, in 0.1 M sodium carbonate buffer (pH 9.5). Coated plates were probed with patient serum (6 morphea and 6 multiple sclerosis) diluted 1:200 in assay diluent. After several washes, the plates were subsequently incubated with horseradish peroxidase-conjugated goat anti-human IgG secondary antibodies (Chemicon). Colorimetric readings were obtained using an ELISA microplate reader (λ = 450; Biotek Instruments, Winooski, VT). Positive serum autoantibody reactivity was defined as values that were greater than the mean of the healthy control sera plus 2 standard errors.

### Histopathology

Formalin-fixed paraffin-embedded 5 µm tissue sections were stained by hematoxylin and eosin (H&E). Morphea biopsies were grouped into inflammatory, mixed (inflammatory and sclerotic), or sclerotic according to criteria by Gilmour et al. [19] and slightly modified by Dhaliwal et al. [20]. The intensity of dermal inflammation was categorized as absent (score = 0), mild (score = 1), moderate (score = 2), or dense (score = 3) according to previously described criteria [20, 21]. The control group was collected from consecutive biopsies of other skin conditions, which included discoid lupus erythematosus, granuloma annulare, psoriasis, lichen planus, and mycosis fungoides.

### Detection and scoring of perineural inflammation

Myelin basic protein immunohistochemistry staining was used in all morphea and control cases to improve detection of peripheral nerves. Immunohistochemistry was performed on 4 µm thick formalin-fixed paraffin-embedded tissues (multi-tumor sandwich blocks and morphea tissue). Tissue sections were deparaffinized in xylene and rehydrated. Endogenous peroxidase activity was quenched at room temperature followed by heat induced epitope retrieval using standard protocols. Slides were immersed in preheated retrieval solution, allowed to cool to room temperature, and then washed with PBS and de-ionized water. Primary antibody incubation was performed using mouse monoclonal anti-MBP (1:100 dilution; Clone 7H11, Antibody ID AB_563893, Cat #NCL-MBP, Leica Biosystems) at room temperature for 50 min. Sections were washed with PBS and then incubated with anti-mouse horseradish peroxidase-conjugated polymer (PowerVision Poly-HRP anti- Mouse IgG, Leica) for 45 min at 25 °C. The slides were then immersed for 8 min in 25 °C diaminobenzidine (DAB) (Invitrogen), counterstained in hematoxylin, and dehydrated.

Any degree of inflammation whereby inflammatory cells surrounded and juxtaposed the perineurium of a nerve was regarded as perineural inflammation. A perineural inflammation index as previously described [20] was used to semi-quantitatively analyze perineural inflammation when detected. Perineural inflammation was assessed and scored as follows: 0 = absent; 1 = occupying < 25% of one high-power field (HPF); 2 = occupying 25–50% of one HPF; 3 = occupying > 50% of one HPF (× 400; field diameter 0.505 mm). Assessment was restricted to within a × 200 field of an area that showed perineural inflammation.

### Immunofluorescence

Immunofluorescence was performed on frozen sections of 12 µm thick mouse brain tissue. Tissue sections were rehydrated followed by heat induced antigen retrieval. Blocking was performed using normal goat serum for 1 h. Primary antibody staining was performed using human serum (4 MBP positive human sera and 4 MBP negative human sera) and mouse anti-myelin basic protein (1:500; #NE1019, Millipore, Temecula, CA) and incubated overnight at 4 °C. Sections were washed with PBS then incubated with goat anti-human Alexa Fluor 488 labeled secondary antibody (#A-11013) and goat anti-mouse Alexa Fluor 594 labeled secondary antibody (#A-11032, both 1:500; ThermoFisher). After washing with PBS, sections were incubated with DAPI (1 µγ/ml) for 10 min. Slides were coverslipped using Aqua-Poly/Mount mounting media (Polysciences, Warrington, PA). Images were taken using a confocal microscope (Zeiss LSM 880) using 20X and 63X objectives.

### Statistics

We applied the Significance Analysis of Microarrays (SAM) algorithm [22] and generated binding reactivity heatmaps using Multiexperiment Viewer (MeV TM4 Microarray Software Suite, version 10.2; Dana-Farber Cancer Institute) using average linkage Euclidean distance hierarchical clustering as described previously [23].

Cut off values for determination of differential autoantigen reactivity between cases and controls were set at a fold change > 1.9, false discovery rate (FDR) of < 0.05 and p-value < 0.01. The unsupervised hierarchical cluster algorithm embedded within the MEV TM4 Microarray Software Suite was used to stratify subjects in the SAM identified list based on autoantibody reactivity profile similarity. For the protein array data, positive serum autoantibody reactivity was designated as values that were greater than the mean of the healthy control sera plus two standard errors. Receiver operating characteristic (ROC) analysis were utilized to confirm the above cutoff values and classification evaluation metrics of precision, recall and F1-score were calculated. To examine whether the proportion of autoantibodies was different between patients with morphea and controls, the two-tailed χ^2^ or Fisher exact test was used. Mann–Whitney *U* test was used to examine if there were significant differences in clinical scores between patients with morphea with or without autoantibodies. In order to determine whether there were significant differences in titers of MBP peptides between healthy controls, multiple sclerosis patients, and morphea patients, the Kruskal–Wallis test was used. In all cases, *P* < 0.05 was considered statistically significant. GraphPad Prism 8 statistical software and R version 3.6.2 were used for all analyses.

## Results

### Patient recruitment

We identified 70 morphea patients with active, inflammatory skin lesions along with 35 matched healthy controls and 30 systemic sclerosis (SSc) disease controls to obtain seroprofiles on autoantigen arrays (Additional file 1: Table S1).

### Protein array analysis identified a distinct biosignature in morphea patients dominated by myelin basic protein (MBP) antibodies

**Fig. 1:**
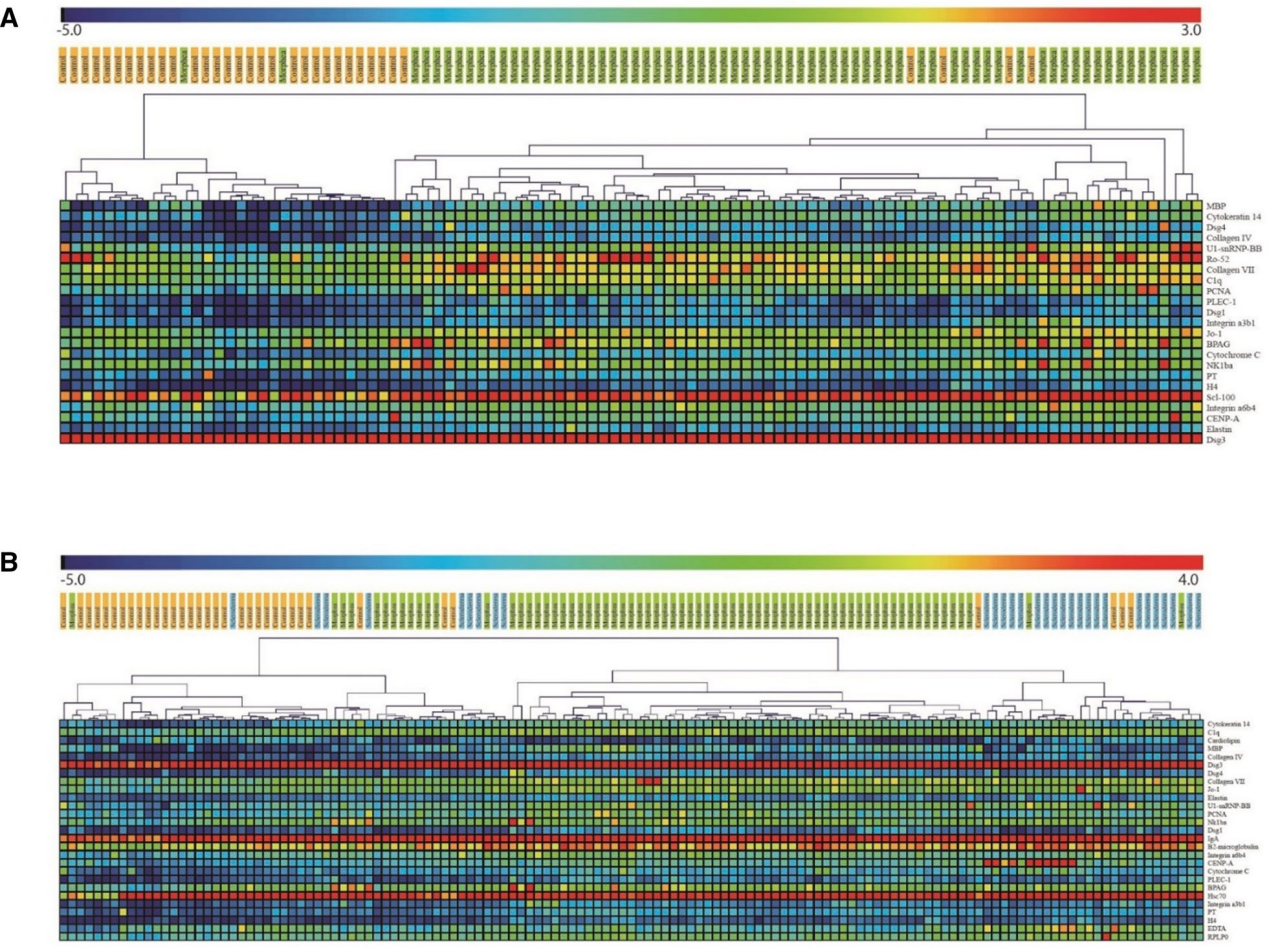
Antigen array profiling identifies a distinct autoantibody biosignature in morphea sera. Heatmaps displaying significant serum IgG reactivity associated with (**A**) morphea cases versus healthy controls or (**B**) morphea cases versus disease (scleroderma) controls versus healthy controls. Autoimmune microarrays were incubated with diluted sera, autoantibody binding was detected with fluorescently-labeled anti-human IgG secondary antibody, and arrays were scanned to quantify binding to each antigen feature. SAM analysis identified antigen features with statistical differences in reactivity (*q* < 0.01). Red represents high reactivity, green intermediate reactivity, and blue lack of reactivity

**Fig. 2:**
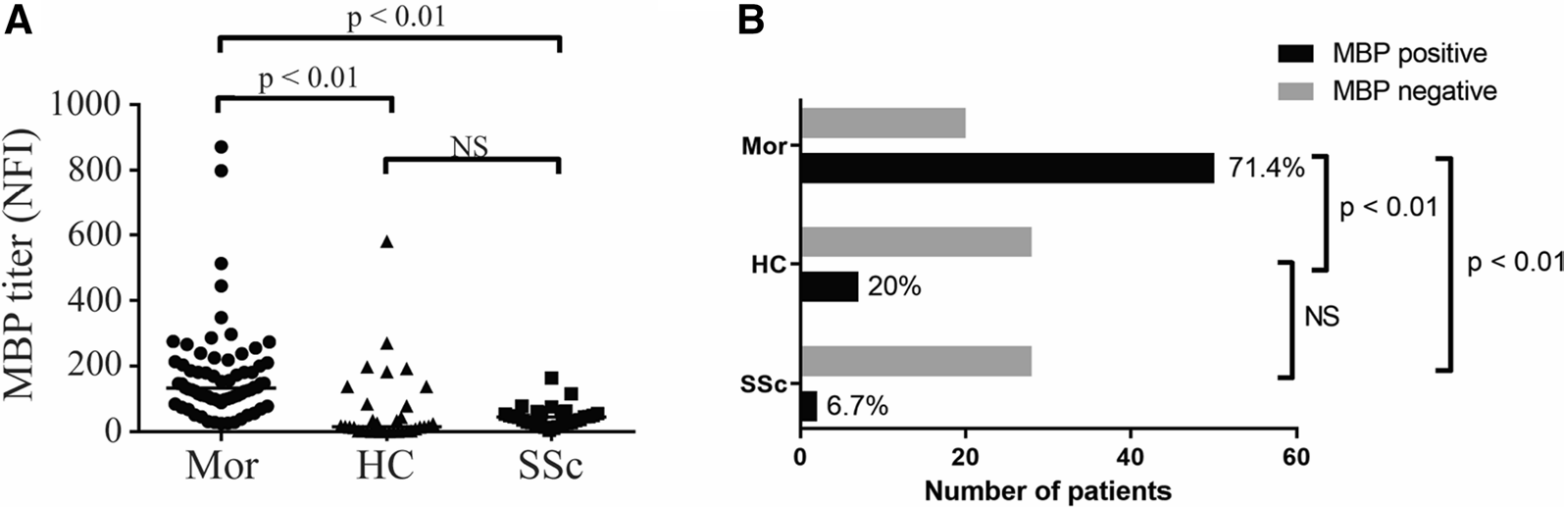
Increased serum MBP autoantigen reactivity in morphea cases compared to controls. **A** Detection of serum MBP autoantigen reactivity by antigen array (net fluorescence intensities, NFI) for morphea cases (Mor), healthy controls (HC) and disease (scleroderma) controls (SSc). **B** Percentage of morphea cases, healthy controls, and disease (scleroderma) controls patients that harbor MBP-positive autoantibodies by antigen array. An individual was considered to be positive for MBP autoantibodies in the respective value was higher than the sum: healthy control mean + 2(standard error). *P* values < 0.05 were considered to be statistically significant

Protein array analysis identifies autoreactive B cell responses in sera from morphea participants. Over 100 proteins representing cutaneous autoantigen epitopes were printed on the autoantigen microarray to determine IgG antibody reactivity in sera. The Significance Analysis of Microarray (SAM) algorithm identified 23 antigens that differentially reacted with the IgG autoantibodies in the sera of morphea patients compared to those of healthy controls (Fig. 1A, Additional file 1: Table S2). These 23 autoantibodies were identified as candidates for additional analysis. Next, we compared autoantibody profiles of morphea patients, SSc disease controls, and healthy controls to determine which autoantibodies were reactive in morphea and SSc. The SAM identified 27 antigens that differentially reacted with the IgG autoantibodies in the sera of morphea patients compared to those of SSc disease controls and healthy controls (Fig. 1B, Additional file 1: Table S3). Using unsupervised hierarchical clustering of the differentially expressed autoantigens by SAM, we determined that systemic sclerosis and morphea autoantibody reactivities exhibited distinct biosignatures that were largely associated with the presence of anti-MBP in morphea, but not in SSc or healthy controls. The morphea cohort exhibited a significantly increased median net fluorescence intensity (NFI) for MBP of 133 compared to 15.5 in matched healthy controls (p < 0.01) and 45.5 in systemic sclerosis patients (p < 0.01) (Fig. 2A). Of all the morphea cases, 71.4% exhibited increased MBP autoantibodies compared to 20% of healthy controls and 6.7% of systemic sclerosis cases by NFI (Fig. 2B). Thus, we sought to further characterize anti-MBP antibodies in morphea. ROC analysis of anti-MBP autoantibodies in morphea patients against healthy controls revealed area under curve (AUC) of 0.836 (0.734–0.937) (Additional file 1: Figure S1). Using a cutoff of 2 standard errors above the mean in healthy controls, we determined good precision (88%), recall (71%), and F1-score (79%) in morphea patients. Morphea patients also exhibited increased Cytokeratin 14 and DSG4 antibodies compared to healthy controls and systemic sclerosis cases by NFI. These antigens are located in the epidermis and associated with disorders of the epidermis such as bullous disorders [24, 25]. Because the pathology of morphea is dermal, we chose to focus on MBP antibodies given the antigen’s dermal location. The identification of autoantibodies against centromere, U1-snRNP-bb, and Ro-52 in the systemic sclerosis group was important because these are known to be associated with systemic sclerosis [26]. Although DSG3 autoantibodies were found to be expressed in all three groups, the fold-change between groups was smaller than the rest of the proteins identified by SAM analysis. Furthermore, confirmatory ELISA for the presence of DSG3 antibodies in the sera of morphea patients was negative (data not shown) [27].

### Increased perineural inflammation observed in skin biopsies from inflammatory morphea lesions

**Fig. 3:**
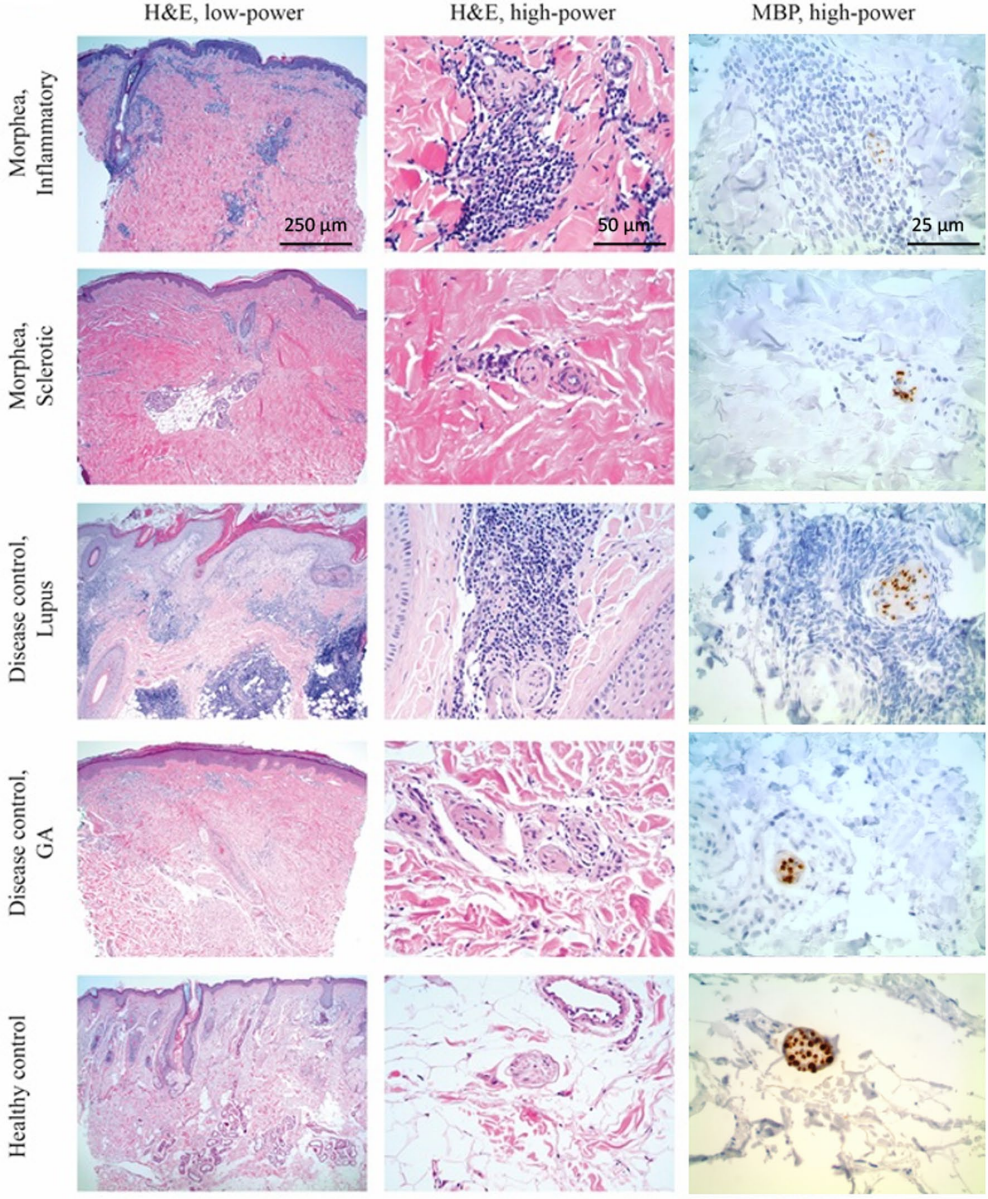
Immunohistochemistry highlights perineural inflammation in morphea tissue. Representative immunohistochemistry images of sections from inflammatory morphea, sclerotic morphea, discoid lupus erythematosus, granuloma annulare (GA), and healthy tissue. Sections were stained with hematoxylin and eosin (H&E) and images shown in low- (× 40 original magnification) and high-power (× 200). Sections were also stained with antibodies against myelin basic protein (MBP), which highlights peripheral nerves (× 400)

Given the observed autoreactivity to MBP in morphea cases, we aimed to determine whether inflammation was present in the perineural area of morphea skin lesions, which likely contributes to clinical symptoms of the disease such as pain [5, 20]. Representative images of skin tissue sections from inflammatory morphea highlight perineural inflammation (Fig. 3). H&E staining demonstrated increased inflammation surrounding peripheral nerves in inflammatory morphea compared to granuloma annulare and healthy tissue controls. Immunohistochemistry with antibodies against MBP further highlighted inflammation surrounding peripheral cutaneous nerves, including small-caliber forms. Quantification of perineural inflammation is shown in Additional file 1: Table S4. Whereas there is an equivalent or higher amount of dermal inflammation in control cases (mean score 2.0) compared to morphea cases (mean score inflammatory morphea 2.0, mixed morphea 1.7, sclerotic morphea 0.6, all morphea 1.3), the degree of perineural inflammation is greater in inflammatory morphea (mean score 1.7), mixed morphea (mean score 1.3), and all morphea (mean score 1.0) compared to control cases (mean score 0.3) (p < 0.05). Discoid lupus erythematosus serves as a positive control given its well-recognized relationship with perineural inflammation [28]. In sclerotic morphea, the amount of both dermal and perineural inflammation was dampened compared to inflammatory or mixed morphea.

### Sera from MBP positive patients stained myelinated areas of mouse brain sections

**Fig. 4:**
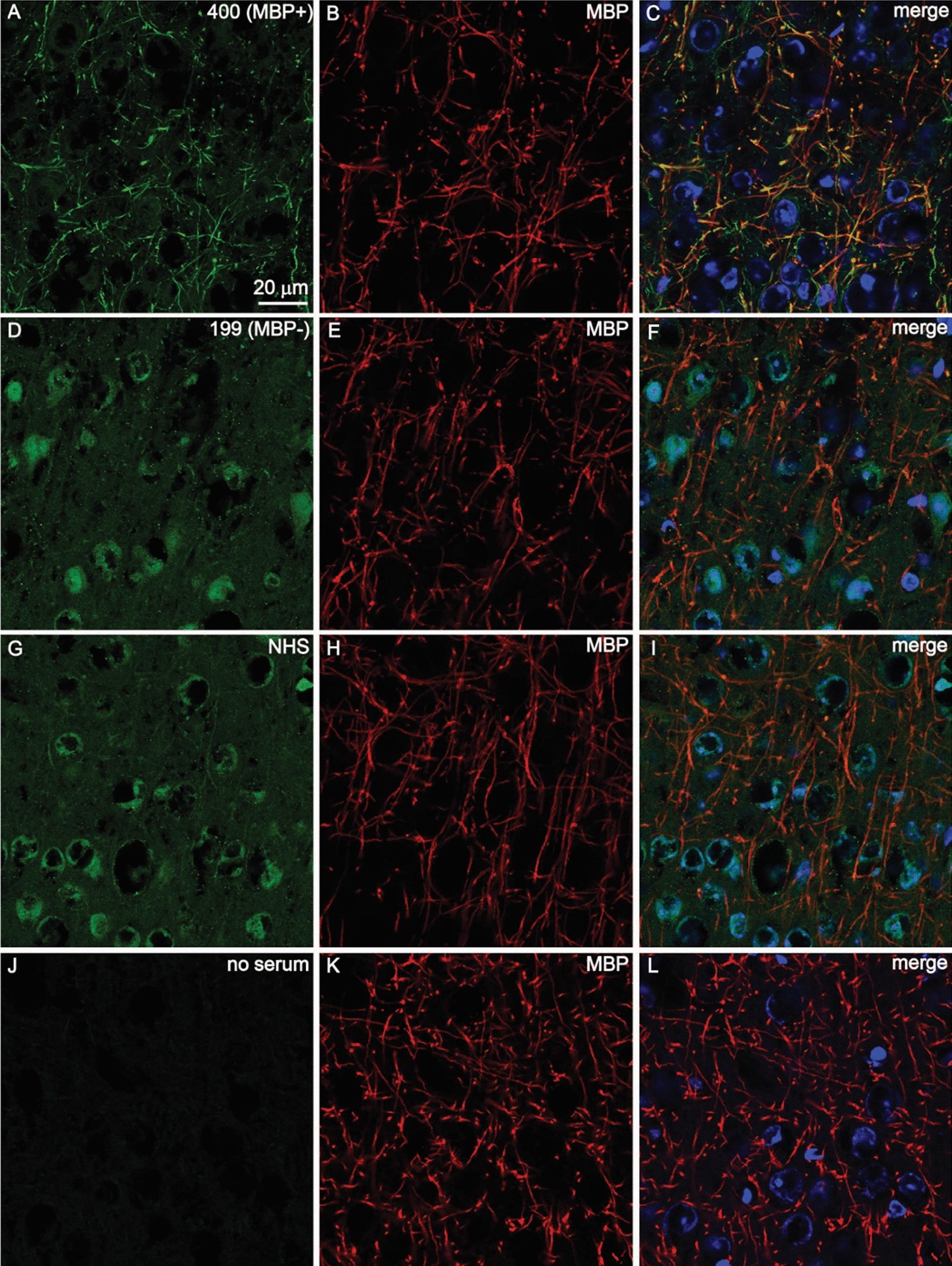
Double immunofluorescent staining of mouse brain cortex with human sera and anti-MBP antibodies. Representative images of cortex sections stained with MBP-positive morphea patient serum 400 (**A**–**C**), MBP-negative morphea patient serum 199 (**D**–**F**), normal human serum (NHS; **G**–**I**) and no human serum (**J**–**L**) together with commercial anti-MBP antibodies. Human serum staining is shown in green (**A**, **D**, **G**, and **J**), and MBP staining is shown in red (**B**, **E**, **H**, and **K**). Merged images (**C**, **F**, **I**, and **L**) show human serum staining, MBP staining, and nuclear staining with DAPI in blue. Images were taken using a 63X objective. Scale bar (20 µm) shown in panel A applies to all images in the figure. Only MBP-positive morphea patient serum 400 shows robust staining in MBP-rich myelinated axons in a similar pattern (**C**) compared to MBP-negative patient serum 199 (**F**), normal human serum (**I**) or no human serum (**L**)

In order to confirm the presence of MBP antibodies in morphea patient sera, we selected patients that were MBP positive and MBP negative by protein microarray. Immunofluorescence analysis showed that MBP positive morphea sera labeled myelinated axons in mouse brain sections as judged by co-staining with a commercial monoclonal antibody directed against MBP. Healthy control sera and MBP negative morphea sera did not show robust fluorescent labeling in MBP-rich areas, confirming the presence of MBP antibodies in morphea patient sera (Fig. 4). Positive immunolabelling of neuronal cell bodies and other non-axonal structures was consistently observed in brain tissue stained with MBP-negative serum samples, indicating the presence of other brain specific antibodies. Indeed, it was observed that in many of the morphea patients in this cohort regardless of MBP antibody positivity, there was positive staining in the neuronal cell bodies and vasculature (Additional file 1: Table S5).

### Morphea patients with anti-MBP antibodies reported pain more frequently

We next determined whether there were any clinical parameters that distinguished morphea patients with high serum titer MBP autoantibodies compared to morphea patients that did not have these serum autoantibodies. As detailed in Table 1, we identified a total of 50 morphea patients with anti-MBP antibodies via protein microarray representing 71.4% of the total samples. There were no differences in the demographic characteristics between groups. However, the percentage of those with MBP autoantibodies that had pain was greater than in those without antibodies, although this failed to reach statistical significance likely due to small sample size.

**Table 1:** Clinical characteristics of morphea participants with MBP autoantibodies

Parameter	MBP-positive	MBP-negative	P value^b^
Total, No (%)	50 (71.4%)	20 (28.5%)	
Age, median (IQR), years	43 (19–63)	41 (25–59)	0.75
Sex, F/M, No	46F/4 M	16F/4 M	0.21
Ethnicity: White, No (%)	36 (72.0%)	16 (80.0%)	0.56
Morphea subtype:			0.14
Generalized, No (%)	24 (48.0%)	11 (55.0%)	
Linear, No (%)	20 (40.0%)	4 (20.0%)	
Plaque, No (%)	4 (8.0%)	5 (25.0%)	
Mixed, No (%)	2 (4.0%)	0 (0%)	
LoSCAT component scores:	n = 25	n = 11	
mLoSSI, median (IQR)	26 (6–48)	11 (9–20)	0.61
LoSDI, median (IQR)	22 (8–30)	10 (7–23)	0.45
Lesional Pain, No (%)^a^			0.39
Yes	19 (38.0%)	5 (25.0%)	1
No	25 (50.0%)	12 (60.0%)	
Lesional Itch, No (%)^a^			
Yes	29 (58.0%)	12 (60.0%)	
No	15 (30.0%)	5 (25.0%)	

IQR, interquartile range, LoSCAT, localized scleroderma cutaneous assessment tool, mLoSSI, modified localized skin severity index, LoSDI, localized scleroderma damage index

^a^Patient-reported itch and pain scores available for 44 of 50 MBP-positive and 17 of 20 MBP-negative morphea patients

^b^Categorical variables compared using Fisher’s Exact test. Continuous variables were compared between clusters using Mann–Whitney test

### Morphea MBP autoantibodies target a distinct epitope compared to multiple sclerosis MBP autoantibodies

**Fig. 5:**
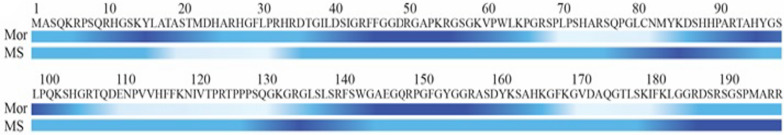
Epitope libraries that span full-length MBP identify overlapping and non-overlapping immunodominant epitopes in morphea cases compared to disease (multiple sclerosis) controls. MBP amino acid sequence in relation to autoantibody reactivity for morphea (Mor) and multiple sclerosis (MS) cases. Higher reactive fragments are displayed in dark blue, less reactive fragments in light blue, and nonreactive fragments in white

Anti-MBP antibodies are typically present in approximately 50% of multiple sclerosis (MS) patients [29]. In an effort to identify whether MBP autoantibodies in morphea cases target similar or distinct epitopes compared to MS cases, we next designed overlapping peptide sequences that spanned the entire distance of the full-length MBP protein. We identified hot spots of reactivity (Fig. 5, Additional file 1: Figure S2) represented by darker blue colors to highlight peptide sequences in which sera demonstrated significant reactivity by ELISA in morphea (data not shown) and MS cases that harbored serum autoantibodies against the full-length MBP protein. Whole MBP and four MBP peptide segments (11–30, 41–60, 51–70, 91–110) had higher reactivity in morphea samples compared to controls (p < 0.05) and also showed no statistically significant difference in reactivity between MS cases and controls (Additional file 1: Table S6). Six MBP peptide segments showed higher reactivity in both morphea and MS samples compared to controls. Lastly, two MBP peptide segments had higher reactivity in MS samples compared controls and showed no difference in reactivity between morphea and controls. Of note, MS samples had higher reactivity compared to controls for whole MBP (p = 0.08) and peptide segment 51–70 (p = 0.06) but were not statistically significant. The MS data agrees with prior MBP epitope mapping reports that have highlighted MBP peptides 51–70, 81–100, and 151–170 as being significant targets in MS [30].

## Discussion

In this report, we describe the application of cutaneous antigen microarrays to profile sera from participants with morphea. Our objective was to identify autoantibody reactivities associated with morphea and determine whether the presence of these autoantibodies defines a specific clinical or demographic subset of morphea. Cutaneous antigen array analysis of autoantibodies identified reactivity against multiple autoantigens that have been described previously in the literature, including ssDNA, anti-histone antibodies, anti-human IgG/Rheumatoid factor, anti-U1RNP, and anti-MMP-1. Anti-histone and ssDNA antibodies have been associated with functional impairment in the linear subtype by our group and others [4]; but are present in low frequency, limiting clinical utility [31]. Our results demonstrated B-cell reactivity against autoantigens that had not been previously associated with morphea. The morphea autoantibody biosignatures were distinct from healthy controls and systemic sclerosis by protein array.

MBP autoantibodies had the highest fold-change using protein microarray and were found in 27.1% of morphea cases (Additional file 1: Table S2). Subsequent ROC analysis and classification evaluation metrics demonstrated good sensitivity, specificity and F1-score for anti-MBP autoantibodies in morphea patients relative to healthy controls. However, antibodies against MBP are also prevalent in patients with Multiple Sclerosis. Thus, we used epitope mapping to determine if antibodies from morphea patients bound MBP epitopes distinct from MS patient antibodies. Indeed, target epitopes for MBP autoantibodies in morphea are distinct from those reported in MS [30]. Most notably, the immunodominant MBP epitopes in morphea included fragments 11–30, 41–60, 91–110, and 141–160. The encephalitogenic MBP peptide 81–103 implicated in the pathogenesis of MS was not a target of antibodies in morphea patient sera, but was a primary target of antibodies in MS patient sera. These data support that while both morphea patients and MS patients harbor antibodies to MBP, the epitopes to which they bind are distinct. This raises the possibility that antibody reactivity to particular MBP epitopes may be used as a biomarker of disease. This data also supports the possibility that reactivity to particular MBP epitopes may elicit particular disease pathology mechanisms. Caution should be taken here as it is important to note that MMP activity can influence MBP proteolysis and decreased activity of MMP has been noted in the development of sclerosis in morphea [31–33]. Thus, it is possible that the MBP epitopes available in morphea patients to elicit an immune response is simply different from those of MS patients. The result would be a distinction in the MBP epitopes recognized by antibodies from morphea versus MS patients and not necessarily an enrichment of antibodies against particular MBP epitopes driven by a particular pathology.

In contrast to MS, morphea is a disorder of the skin and soft tissues. Therefore, we were interested in studying MBP expression in relation to inflammation in skin by immunohistochemistry. Perineural inflammation in morphea is not well described. While peripheral nerve fibers are often detectable by routine histology, we used MBP immunohistochemistry to highlight otherwise difficult-to-detect small-caliber nerve fibers and demonstrate the relationship of MBP-expressing fibers to inflammation. Inflammation was more prominent in early morphea than sclerotic or mixed cases when compared to controls. This data provides strong support that autoreactive B-cell responses among morphea patients are associated with differences in disease manifestations.

Inflammatory pathways directed against MBP can initiate neuropathic pain [34]. Although pain is transmitted by C-nociceptive (not myelinated) and Aδ-afferent fibers (thin myelin cover), damage to the peripheral nervous system causes myelinated Aβ-afferents to also transmit pain [35]. Furthermore, T-cell mediated autoimmune neuropathies, including the rat model for Guillain–Barre syndrome (GBS), induced by immunization with immunodominant MBP peptides causes pain [36]. The MBP positive morphea group more frequently reported pain sensation in morphea lesions and overall bodily pain compared to those found to be MBP negative. This suggests a relationship between MBP autoantibodies, cutaneous inflammation, and symptoms associated with morphea. Active, or inflammatory, morphea lesions are characterized by peripheral erythema and patient-reported increased hypersensitivity to sensation in their lesion [37].

A limitation of the present study is that the number of antigens represented on the cutaneous antigen arrays do not represent all peptides and proteins present in skin. In addition to the results described herein, it is likely that additional antigens expressed in skin are targeted in morphea. Further studies are warranted to determine whether MBP antibodies, or other autoantibodies are present in sera prior to the clinical development of morphea. Additionally, another limitation of this study is the use of linear peptides in the identification of potentially conformational epitopes.

The presented findings suggest that a molecular classification of morphea based on distinct autoantibody biosignatures may represent a promising approach to differentially classify morphea. Our study has identified anti-MBP as a novel antibody associated with morphea due to its increased expression in morphea. Future studies with validation cohorts should be performed to elucidate the pathogenesis of MBP autoantibodies in the skin and causes of pain associated with morphea.

## Supplementary Information

**Additional file 1.** Table S1. Demographics and baseline clinical characteristics of study cohort. Table S2. Antigens targeted by differentially expressed IgG autoantibodies in morphea versus healthy controls in order of fold-change. Table S3. Antigens targeted by differentially expressed IgG autoantibodies in morphea versus healthy controls vs systemic sclerosis disease controls. Table S4. Increased perineural inflammation in morphea cases compared to healthy controls. Table S5. Increased axonal staining of mouse brain sections by MBP positive morphea sera compared to MBP negative sera. Table S6. Summary of anti-MBP peptide biosignature in morphea. Figure S1. ROC Curve analysis of MBP. 95% confidence intervals are plotted and shaded in blue and provided for area under curve (AUC). Figure S2. MBP peptide titers in healthy control, morphea, and multiple sclerosis patients. MBP whole peptide and epitope peptide titers were compared between the three groups using Kruskal-Wallis test. *p < 0.05, **p < 0.01, *** p < 0.001.

## Data Availability

The datasets used and/or analyzed during the current study are available from the corresponding author on reasonable request.

## Abbreviations

AUC: Area under curve
DLE: Discoid lupus erythematosus
DLQI: Dermatology Life Quality Index
GBS: Guillain–Barre syndrome
GENISOS: Genetic versus Environment in Scleroderma Outcomes Study
LoSCAT: Localized Scleroderma Cutaneous Assessment Tool
LoSDI: Localized Scleroderma Damage Index
mLoSSI: Modified Localized Skin Severity Index
MAC: Morphea in Adults and Children Cohort
MMP: Matrix metalloproteinase
MBP: Myelin basic protein
MeV: Multiexperiment viewer
MS: Multiple sclerosis
PGA: Physician’s global assessment
PRS: Parry Romberg Syndrome
SAM: Significance analysis of microarrays
SSc: Systemic sclerosis
