# Supplementary material for: Autoantigen microarrays reveal myelin basic protein autoantibodies in morphea
Source: J Transl Med. 2022 Jan 24;20:41. doi: 10.1186/s12967-022-03246-5 (PMC8785566; doi:10.1186/s12967-022-03246-5)
Supplement: Supplementary file 1 — Additional file 1. Table S1. Demographics and baseline clinical characteristics of study cohort. Table S2. Antigens targeted by differentially expressed IgG autoantibodies in morphea versus healthy controls in order of fold-change. Table S3. Antigens targeted by differentially expressed IgG autoantibodies in morphea versus healthy controls vs systemic sclerosis disease controls. Table S4. Increased perineural inflammation in morphea cases compared to healthy controls. Table S5. Increased axonal staining of mouse brain sections by MBP positive morphea sera compared to MBP negative sera. Table S6. Summary of anti-MBP peptide biosignature in morphea. Figure S1. ROC Curve analysis of MBP. 95% confidence intervals are plotted and shaded in blue and provided for area under curve (AUC). Figure S2. MBP peptide titers in healthy control, morphea, and multiple sclerosis patients. MBP whole peptide and epitope peptide titers were compared between the three groups using Kruskal-Wallis test. *p < 0.05, **p < 0.01, *** p < 0.001. [file 12967_2022_3246_MOESM1_ESM.docx]

**Table S1.** Demographics and baseline clinical characteristics of study cohort.

| **Characteristic** | **Morphea (N=70)** | **Control (N=35)** | **Systemic Sclerosis (N=30)** |
| --- | --- | --- | --- |
| Sex, n (%) |  |  |  |
| Female | 62 (88.6%) | 31 (88.6%) | 30 (100%) |
| Male | 8 (11.4%) | 4 (11.4%) | 0 (0%) |
| Race/Ethnicity, n (%) |  |  |  |
| White | 52 (74.3%) | 29 (82.9%) |  |
| Black | 2 (2.9%) | 0 (0%) |  |
| Hispanic  Asian  Other  Age, median (IQR)  Morphea subtype, n (%)  Generalized  Linear  Plaque  Mixed  LoSCAT scores^a^  mLoSSI (activity)  LoSDI (damage) | 14 (20%)  1 (1.4%)  1 (1.4%)  43.5 (23-60.5)  35 (50%)  26 (37.1%)  9 (12.9%)  2 (2.9%)  17 (6-46)  13 (8-30) | 4 (11.4%)  1 (2.9%)  0 (2.9%))  47 (31-58)  N/A  N/A | 46 (29-64)  N/A  N/A |
| ^a^LoSSI scores available for 35 of morphea patients.  Abbreviations: IQR, interquartile range, LoSCAT, localized scleroderma cutaneous assessment tool, mLoSSI, modified localized skin severity index, LoSDI, localized scleroderma damage index. | | | |

**Table S2. Antigens targeted by differentially expressed IgG autoantibodies in morphea versus healthy controls in order of fold-change.**

| **Antigen ID** | **Fold Change** | **P-value** | **FDR** |
| --- | --- | --- | --- |
| MBP | 4.8 | 2.3 x 10^-8^ | 2.3 x 10^-6^ |
| Cytokeratin 14 protein (Human) | 4.0 | 1.7 x 10^-13^ | 1.8 x 10^-11^ |
| DSG4 (531-630) protein | 2.9 | 9.5 x 10^-13^ | 9.7 x 10^-11^ |
| Collagen IV | 2.5 | 7.9 x 10^-11^ | 8.1 x 10^-9^ |
| U1-snRNP-BB' | 2.5 | 2.4 x 10^-8^ | 2.5 x 10^-6^ |
| Ro-52 (SSA) | 2.4 | 4.1 x 10^-6^ | 4.2 x 10^-4^ |
| RHCol (Collagen VII) | 2.4 | 5.6 x 10^-12^ | 5.8 x 10^-10^ |
| C1q | 2.4 | 1.3 x 10^-14^ | 1.3 x 10^-12^ |
| PCNA | 2.3 | 9.3 x 10^-10^ | 9.6 x 10^-8^ |
| E.coli | 2.3 | 6.4 x 10^-10^ | 6.6 x 10^-8^ |
| DSG 1 Protein | 2.3 | 7.8 x 10^-9^ | 8.1 x 10^-7^ |
| PLEC 1 (4383-4493) protein | 2.3 | 1.2 x 10^-7^ | 1.2 x 10^-5^ |
| rhIntegrin a 3/b1/VLA-3 | 2.2 | 4.0 x 10^-8^ | 4.1 x 10^-6^ |
| JO-1 | 2.2 | 3.3 x 10^-12^ | 3.4 x 10^-10^ |
| BPAG | 2.2 | 2.1 x 10^-7^ | 2.1 x 10^-5^ |
| Cytochrome C | 2.1 | 1.2 x 10^-7^ | 1.3 x 10^-5^ |
| NK1ba | 2.1 | 4.4 x 10^-8^ | 4.5 x 10^-6^ |
| Human PT | 2.0 | 1.6 x 10^-7^ | 1.6 x 10^-5^ |
| rhIntegrin a 6(x1)b4 | 1.9 | 2.5 x 10^-8^ | 2.6 x 10^-6^ |
| H4 | 1.9 | 1.4 x 10^-7^ | 1.5 x 10^-5^ |
| Elastin | 1.9 | 1.1 x 10^-10^ | 1.1 x 10^-8^ |
| PM/Scl-100 | 1.9 | 1.6 x 10^-6^ | 1.7 x 10^-4^ |
| rhDesmoglein-3/Fc Chimera | 1.9 | 2.7 x 10^-12^ | 2.8 x 10^-10^ |

**Table S3. Antigens targeted by differentially expressed IgG autoantibodies in morphea versus healthy controls vs systemic sclerosis disease controls.**

|  | **Antigen ID** | **Score (d)** | **P-value** | **FDR** |
| --- | --- | --- | --- | --- |
| 1 | Cytokeratin 14 protein (Human) | 1.6 | 9.7 x 10^-14^ | 9.9 x 10^-12^ |
| 2 | C1q | 1.5 | 6.8 x 10^-15^ | 7.0 x 10^-13^ |
| 3 | Cardiolipin | 1.4 | 1.5 x 10^-14^ | 1.5 x 10^-12^ |
| 4 | MBP | 1.3 | 2.5 x 10^-12^ | 2.6 x 10^-10^ |
| 5 | Collagen IV | 1.2 | 3.2 x 10^-11^ | 3.3 x 10^-9^ |
| 6 | rhDesmoglein-3/Fc Chimera | 1.2 | 4.9 x 10^-13^ | 5.1 x 10^-11^ |
| 7 | DSG4 (531-630) protein | 1.2 | 1.3 x 10^-13^ | 1.3 x 10^-11^ |
| 8 | RHCol | 1.1 | 3.4 x 10^-12^ | 3.5 x 10^-10^ |
| 9 | JO-1 | 1.0 | 6.8 x 10^-12^ | 7.0 x 10^-10^ |
| 10 | Elastin | 1.0 | 4.6 x 10^-11^ | 4.8 x 10^-9^ |
| 11 | E.coli | 1.0 | 8.7 x 10^-10^ | 9.0 x 10^-8^ |
| 12 | U1-snRNP-BB | 0.94 | 1.5 x 10^-8^ | 1.5 x 10^-6^ |
| 13 | PCNA | 0.92 | 2.1 x 10^-9^ | 2.2 x 10^-7^ |
| 14 | NK1ba | 0.91 | 2.9 x 10^-9^ | 3.0 x 10^-7^ |
| 15 | DSG 1 Protein | 0.91 | 4.0 x 10^-9^ | 4.1 x 10^-7^ |
| 16 | B2-microglobulin | 0.85 | 3.6 x 10^-8^ | 3.7 x 10^-6^ |
| 17 | rhIntegrin a 6(x1)b4 | 0.84 | 1.0 x 10^-7^ | 1.1 x 10^-5^ |
| 18 | CENP-A | 0.82 | 3.9 x 10^-6^ | 4.0 x 10^-4^ |
| 19 | Cytochrome C | 0.82 | 1.4 x 10^-7^ | 1.5 x 10^-5^ |
| 20 | PLEC 1 (4383-4493) protein | 0.81 | 2.4 x 10^-7^ | 2.5 x 10^-5^ |
| 21 | BPAG | 0.81 | 5.6 x 10^-7^ | 5.7 x 10^-5^ |
| 22 | Hsc70 | 0.80 | 9.8 x 10^-11^ | 1.0 x 10^-8^ |
| 23 | rhIntegrin a 3/b1/VLA-3 | 0.79 | 7.2 x 10^-8^ | 7.4 x 10^-6^ |
| 24 | Human PT | 0.78 | 4.0 x 10^-8^ | 4.1 x 10^-6^ |
| 25 | H4 | 0.77 | 2.9 x 10^-7^ | 2.9 x 10^-5^ |
| 26 | Intaktin EDTA | 0.77 | 3.0 x 10^-7^ | 3.1 x 10^-5^ |
| 27 | Ribosomal phosphoprotein P0 | 0.75 | 4.4 x 10^-6^ | 4.5 x 10^-4^ |

**Table S4**. Increased perineural inflammation in morphea cases compared to healthy controls.

|  |  |  |  |  | **Morphea** | | | |
| --- | --- | --- | --- | --- | --- | --- | --- | --- |
|  |  | **Normal (-)** | **DLE (+)** | **Controls^b^** | **All** | **Inflammatory** | **Mixed** | **Sclerotic** |
|  | Total, No | 2 | 2 | 7 | 20 | 3 | 9 | 8 |
| *Perineural Inflammation* | | |  |  |  |  |  |  |
|  | Total positive (%) | 0 (0%) | 2 (100%) | 2 (28.6%) | 13 (65%) | 3 (100%) | 7 (77.8%) | 3 (37.5%) |
|  | Mean score (st dev) | 0 (0) | 2.5 (0.7) ^a^ | 0.3 (0.5) | 1.0 (0.9) ^a^ | 1.7 (0.6) ^a^ | 1.3 (0.9) ^a^ | 0.4 (0.5) |
| *Dermal inflammation* | |  |  |  |  |  |  |  |
|  | Mean score (st dev) | 1.0 (0) | 2.5 (0.7) | 2.0 (0.6) | 1.3 (0.7) ^a^ | 2.0 (0) | 1.7 (0.5) | 0.6 (0.5) ^a^ |

^a^p < 0.05 vs controls; ^b^Control cases included mycosis fungoides, granuloma annulare, lichen planus, and psoriasis.

Abbreviations: DLE, discoid lupus erythematosus

**Table S5.**  Increased axonal staining of mouse brain sections by MBP positive morphea sera compared to MBP negative sera

|  | **Axons** | **Cell Body** | **Vasculature** |
| --- | --- | --- | --- |
|  |  |  |  |
| **MBP (+)**  25  215  244  400 | +/-  +  +  + | +  -  +  - | -  +  -  - |
| **MBP (-)**  145  199  208  346 | +/-  -  -  +/- | +  +  +  + | -  +  -  - |

Key: +, strong positive; +/-, weakly positive, -, negative

**Table S6.** Summary of anti-MBP peptide biosignature in morphea

|  | **Morphea vs control** | **Multiple Sclerosis vs control** | **Increased activity** |
| --- | --- | --- | --- |
| Whole MBP | + | - | MORPHEA |
| 1-20 | + | + | BOTH |
| 11-30 | + | - | MORPHEA |
| 21-40 | - | - | X |
| 31-50 | + | + | BOTH |
| 41-60 | + | - | MORPHEA |
| 51-70 | + | - | MORPHEA |
| 61-80 | - | - | X |
| 71-90 | - | - | X |
| 81-100 | + | + | BOTH |
| 91-110 | + | - | MORPHEA |
| 101-120 | - | - | X |
| 111-130 | - | - | X |
| 121-140 | - | + | MS |
| 131-150 | + | + | BOTH |
| 141-160 | - | - | X |
| 151-170 | + | + | BOTH |
| 161-180 | - | - | X |
| 171-190 | - | + | MS |
| 181-198 | + | + | BOTH |

**Figure S1. ROC Curve analysis of MBP.** 95% confidence intervals are plotted and shaded in blue and provided for area under curve (AUC).

**
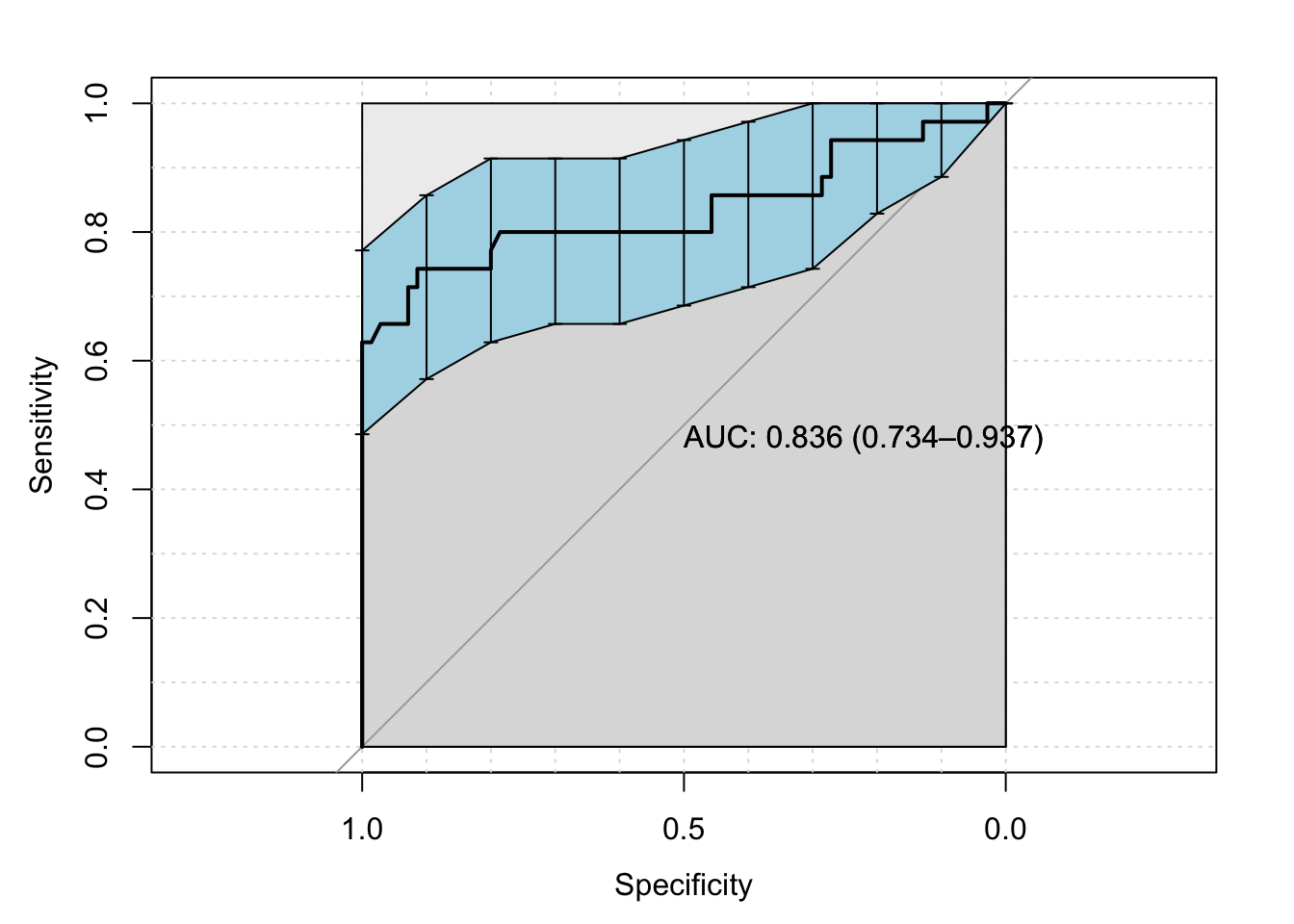
**

**Figure S2. MBP peptide titers in healthy control, morphea, and multiple sclerosis patients.** MBP whole peptide and epitope peptide titers were compared between the three groups using Kruskal-Wallis test. *p < 0.05, **p < 0.01, *** p < 0.001.

**
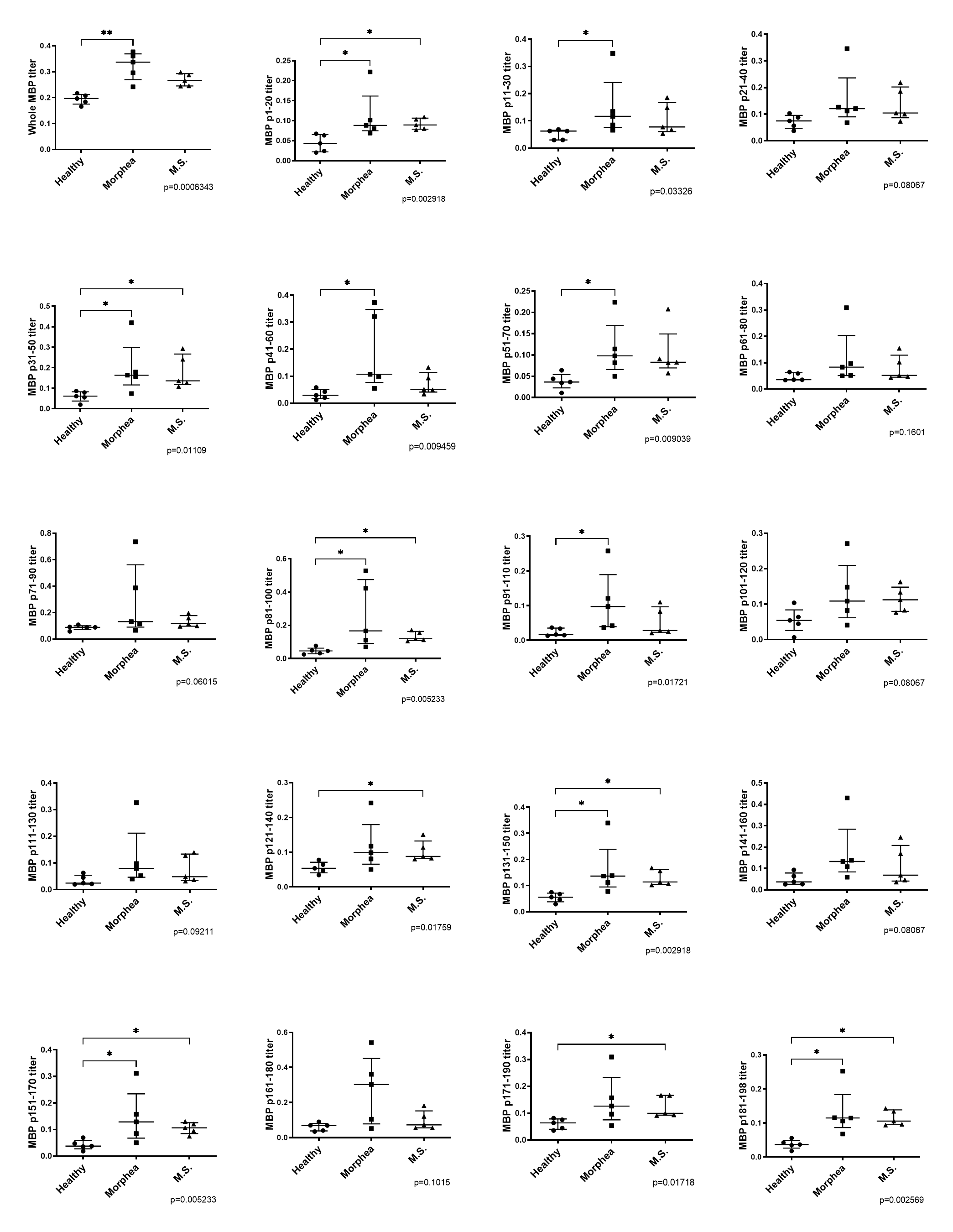
**
